# Supplementary material for: Non-canonical Wnt signaling regulates neural stem cell quiescence during homeostasis and after demyelination
Source: Nat Commun. 2018 Jan 2;9:36. doi: 10.1038/s41467-017-02440-0 (PMC5750230; doi:10.1038/s41467-017-02440-0)
Supplement: Supplementary file 1 — Supplementary Information [file 41467_2017_2440_MOESM1_ESM.pdf]

Supplementary Figure 1

a Proteins encoded by genes enriched in qNSCs

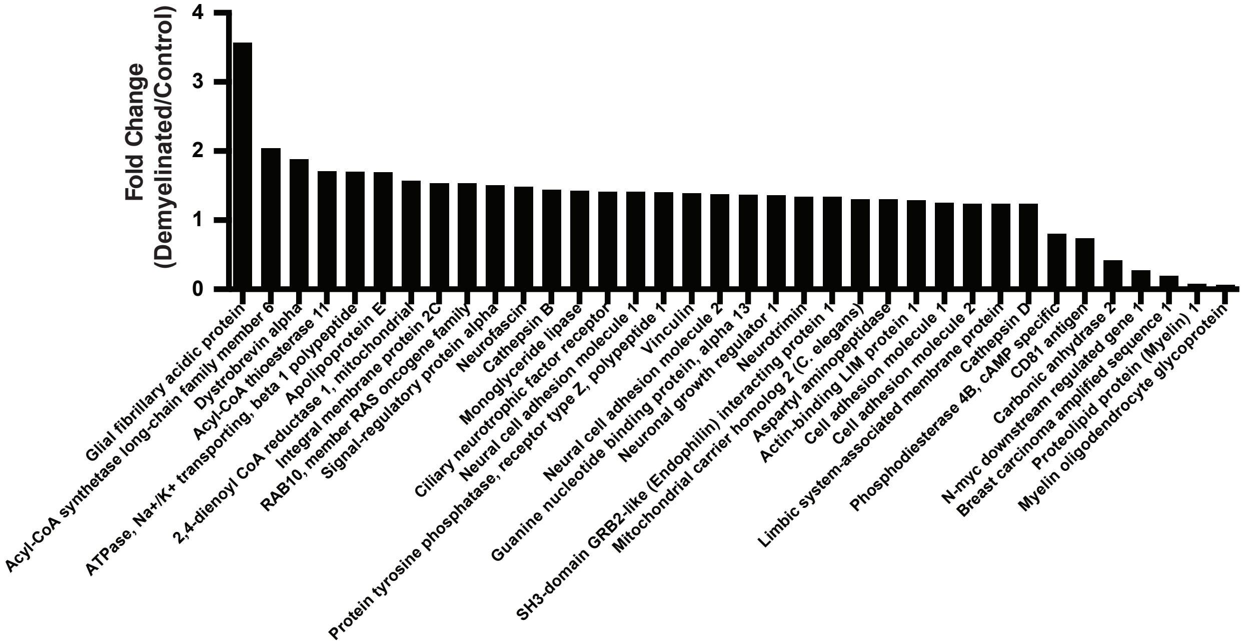

b

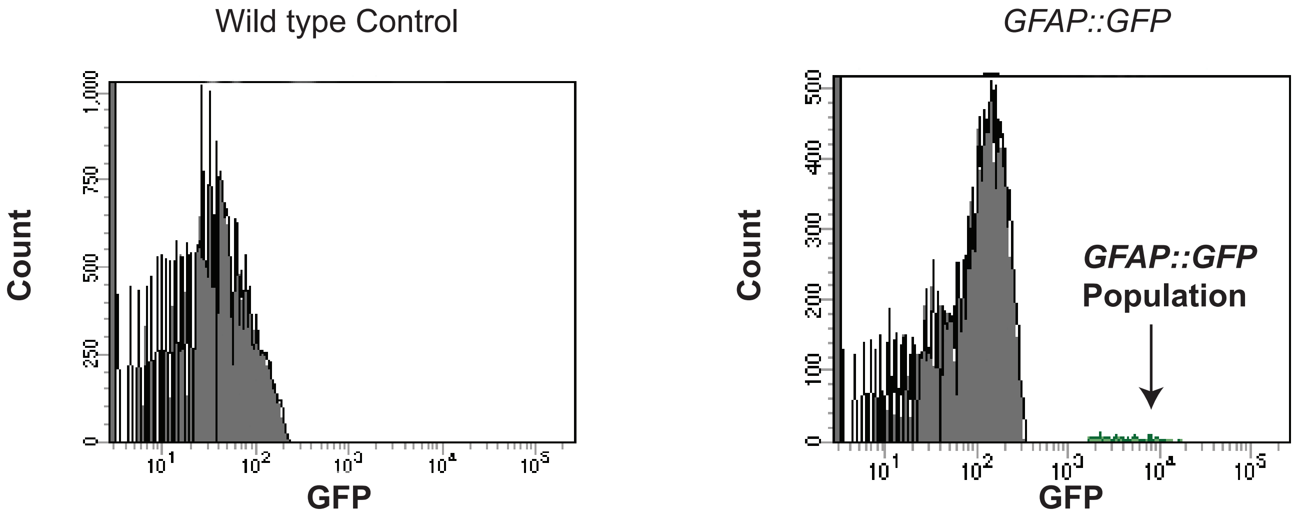

c

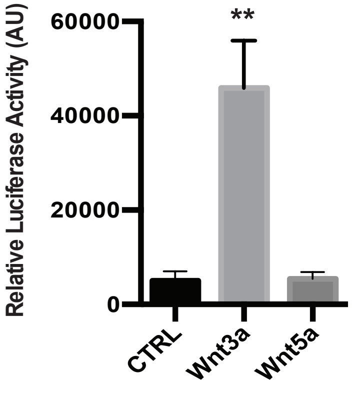

Supplementary Figure 1 Additional Analysis of Proteomics and Wnt Stimulation Assays.

(a) Proteins encoded by genes that were shown to be enriched in quiescent neural stem cells (see Codega et al., [5]) and identified in the SVZ proteomics screen after a demyelinating injury.

(b) Typical FAC-sorting plots of *GFAP::GFP*$^{+}$ NSCs that were collected for analyzing mRNA levels of Wnt co-receptors.

(c) Verification of Wnt conditioned media using a TCF/LEF HEK293 reporter cell line. Canonical Wnt3a induces a high luciferase activity, whereas non-canonical Wnt5a does not induce any activity, confirming that it acts in a TCF/LEF independent (non-canonical) manner. **$p<0.01$; error bars represent mean±s.e.m. n=3 independent experiments for (c).

Supplementary Figure 2

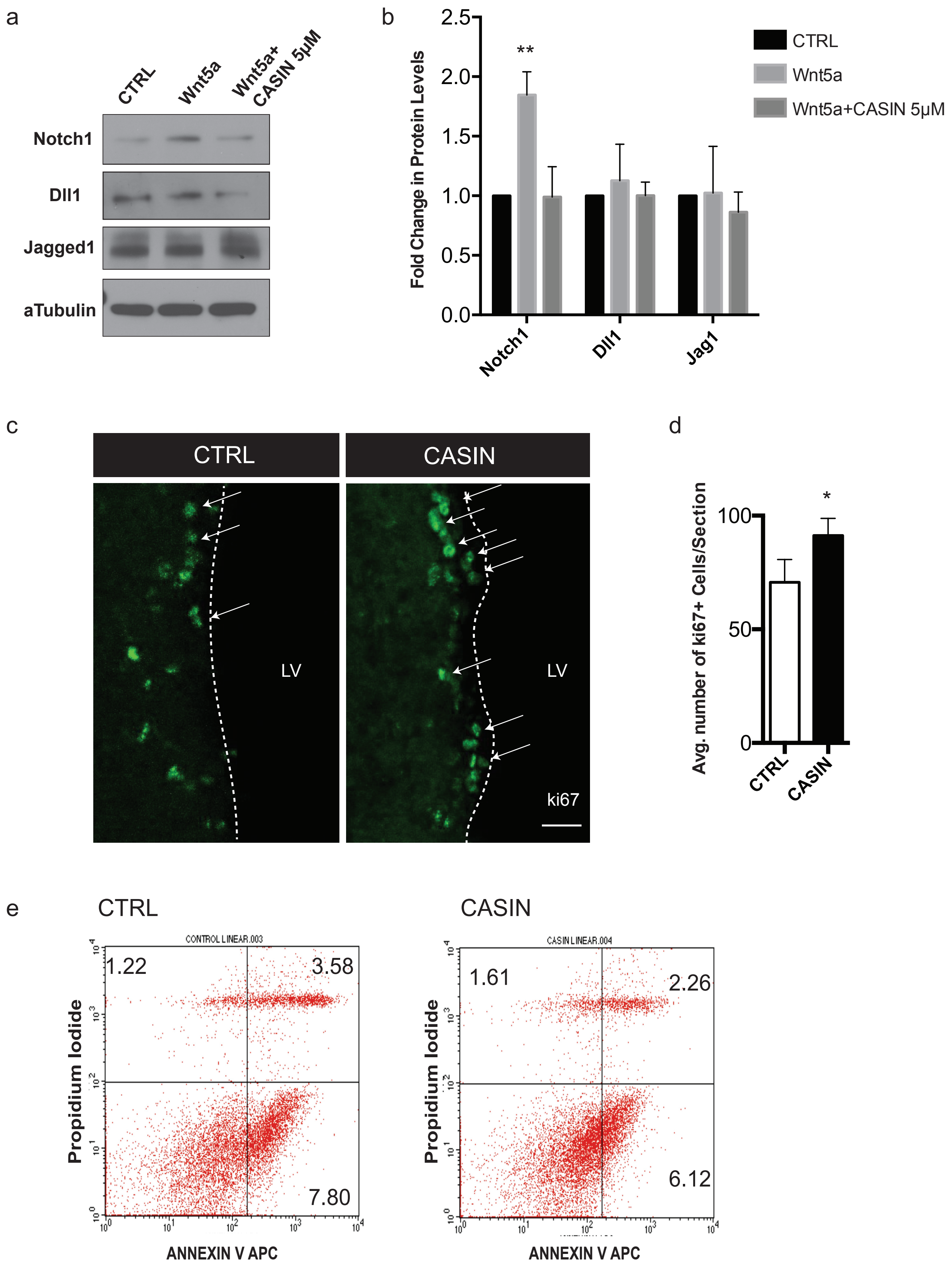

Supplementary Figure 2 Additional Analysis of Wnt5a Induced Non-canonical Wnt Signaling and Cdc42 Activity in SVZ NSCs

(a) Protein expression of Notch1, Dll1, and Jagged1 in SVZ explants after Wnt5a stimulation. It can be noted that Wnt5a induces expression of Notch1 in a Cdc42 dependent manner, but not Dll1 or Jagged1.

(b) Quantification of blots shown in (a). Protein levels were normalized to alpha tubulin levels and represented as fold change over the control condition.

(c) Representative images of ki67$^+$ cells in the SVZ of CTRL (vehicle) and CASIN injected mice. Note an increase in ki67$^+$ cells in CASIN injected mice. Scale bar represents 50μm.

(d) Quantification of ki67$^+$ cells in the SVZ of CTRL (vehicle) and CASIN treated mice. (Total number of cells counted for CTRL:566; CASIN:1017).

(e) CASIN treatment does not induce cell death in SVZ cells, as detected by flow cytometric analysis of SVZ cells from CTRL and CASIN treated mice labeled with annexin V and propidium iodide. *$p<0.05$, **$p<0.01$; error bars represent mean±s.e.m. n=3 independent experiments for (b, d).

Supplementary Figure 3

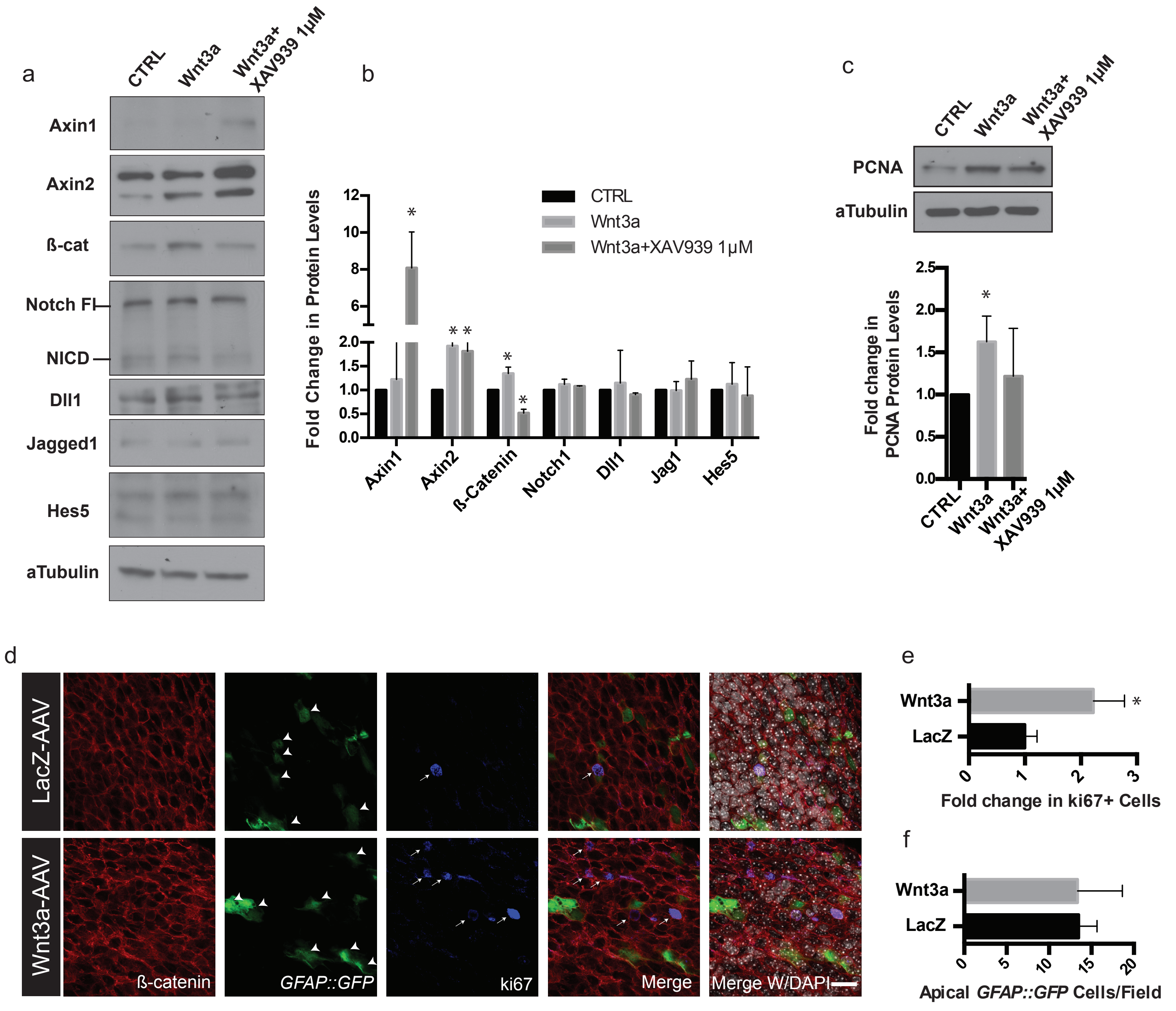

Supplementary Figure 3 Canonical Wnt/ß-Catenin Signaling does not Affect Notch1 Activity but Induces Proliferation in the SVZ

(a) Immunoblots of canonical Wnt and Notch1 signaling effectors and their downstream targets in SVZ explants treated with Wnt3a or Wnt3a+XAV939 1μM.

(b) Quantification of blots shown in (a). Protein levels were normalized to alpha tubulin levels and represented as fold change over the control condition.

(c) Canonical Wnt/ß-catenin signaling promotes cell proliferation as shown by increased proliferating cell nuclear antigen (PCNA) protein levels in SVZ explants stimulated with Wnt3a.

(d) Representative en-face view images of SVZ wholemounts from *GFAP::GFP* mice whose SVZs were infected with LacZ-AAV (top panels) or Wnt3a-AAV (bottom panels) and immunostained for ß-catenin and ki67. Scale bar represents 20μm.

(e-f) Wnt3a induces proliferation as an increase in $ki67^+$ cells can be observed (e). No significant changes in the numbers of apical $GFAP::GFP^+$ cells were observed under the same conditions (f). *$p<0.05$; error bars represent mean±s.e.m. n=3 independent experiments for (b, c, e, f).

Supplementary Figure 4

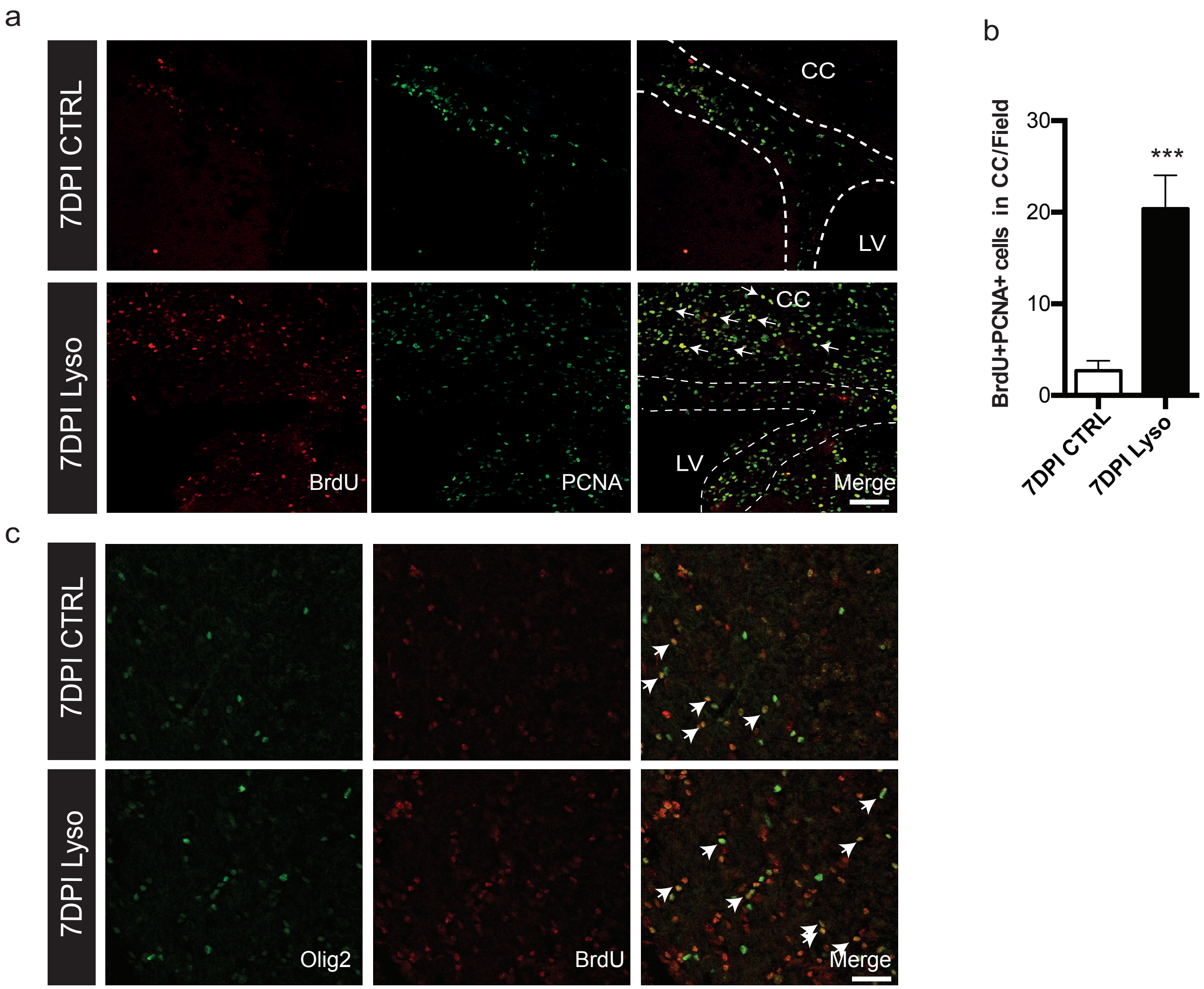

Supplementary Figure 4 Activation and Migration of SVZ NSC Progeny after Demyelination Injury

(a) Representative confocal images at the dorsal region of the SVZ shows diluted $BrdU^{+}PCNA^{+}$ cells migrating out of the SVZ into the corpus callosum (CC). Scale bar represents 50μm.

(b) Quantification of diluted $BrdU^{+}PCNA^{+}$ cells in the corpus callosum.

(c) Increased numbers of diluted $BrdU^{+}$ cells that migrate to the CC after demyelination express Olig2 (indicated by arrows), indicating their progression towards a oligodendrogenic lineage. Scale bar represents 50μm. ***$p<0.001$; error bars represent mean±s.e.m. n=3 independent experiments (b).

Supplementary Figure 5

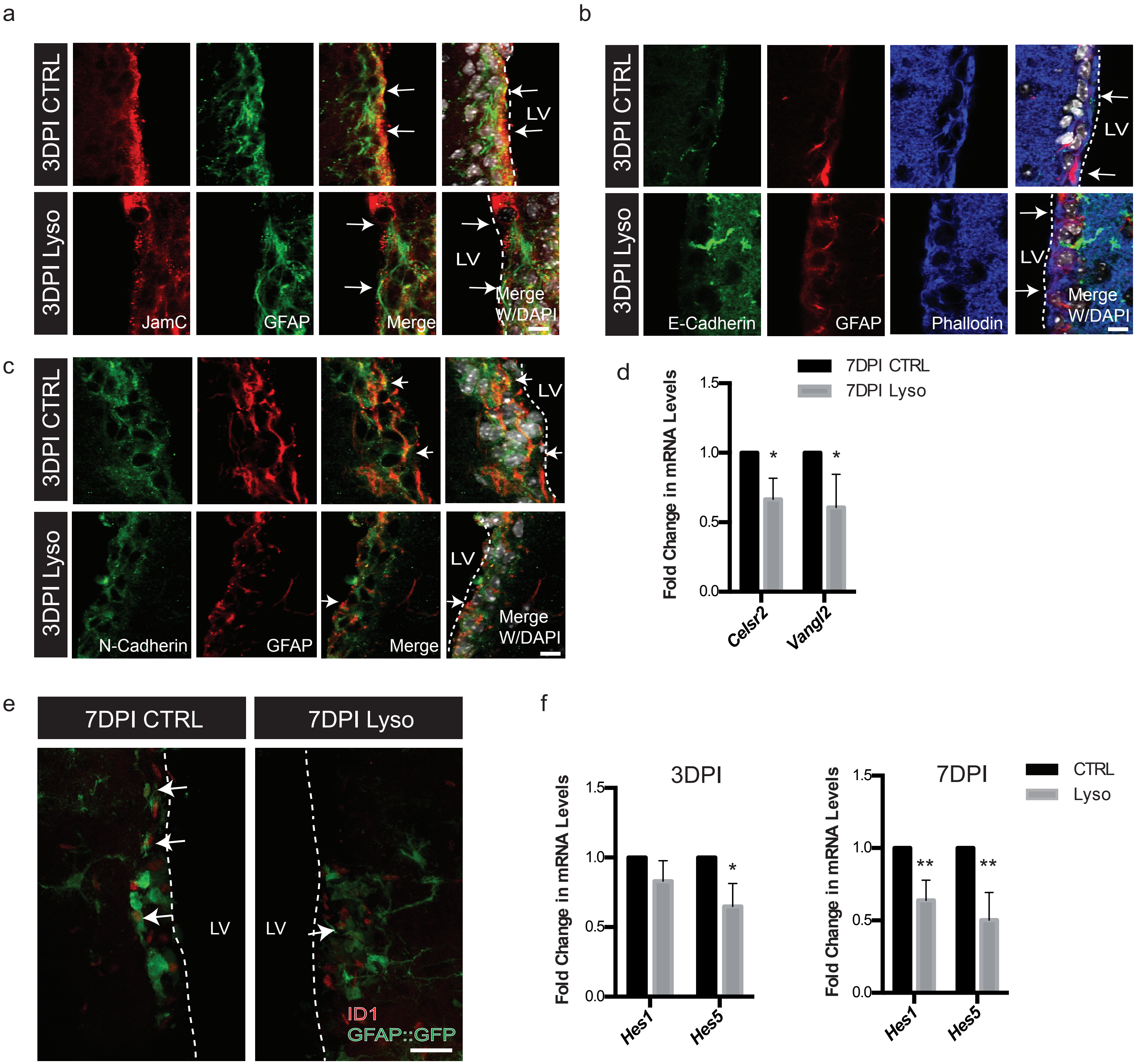

Supplementary Figure 5 Alterations in SVZ Cytoarchitecture and Molecular Signaling During Demyelination

(a-c) Representative confocal images of coronal brain sections showing the apical border (ventricular wall) of the SVZ labeled with anti-Jam-C (a), anti-E-cadherin (b), anti-N-Cadherin (c), and anti-GFAP antibodies. Note the reduction in the staining intensity of Jam-C, E-Cadherin, and N-Cadherin in the $GFAP^{+}$ cells along the apical border of the ipsilateral SVZ at 3DPI. Scale bars in (a-c) represent 20μm.

(d) Transcript levels of *Celsr2* and *Vangl2* in the SVZ at 7DPI show a significant reduction after demyelination.

(e) Representative confocal images of coronal brain sections from demyelinated *GFAP::GFP* mice labeled with anti-Id1 at 7DPI. Note a decrease in *GFAP::GFP*$^{+}$Id1^{+} cell numbers at 7DPI (indicated by arrows). Scale bar represents 20μm.

(f) Transcript levels of *Hes1* and *Hes5* in the SVZ at 3DPI and 7DPI. Transcript levels of each gene at 3DPI and 7DPI are represented as fold change over the control SVZ at the corresponding time point. *$p<0.05$, **$p<0.01$; error bars represent mean±s.e.m. n=3 for (d), n=6 for (f); n= independent experiments.

Supplementary Figure 6

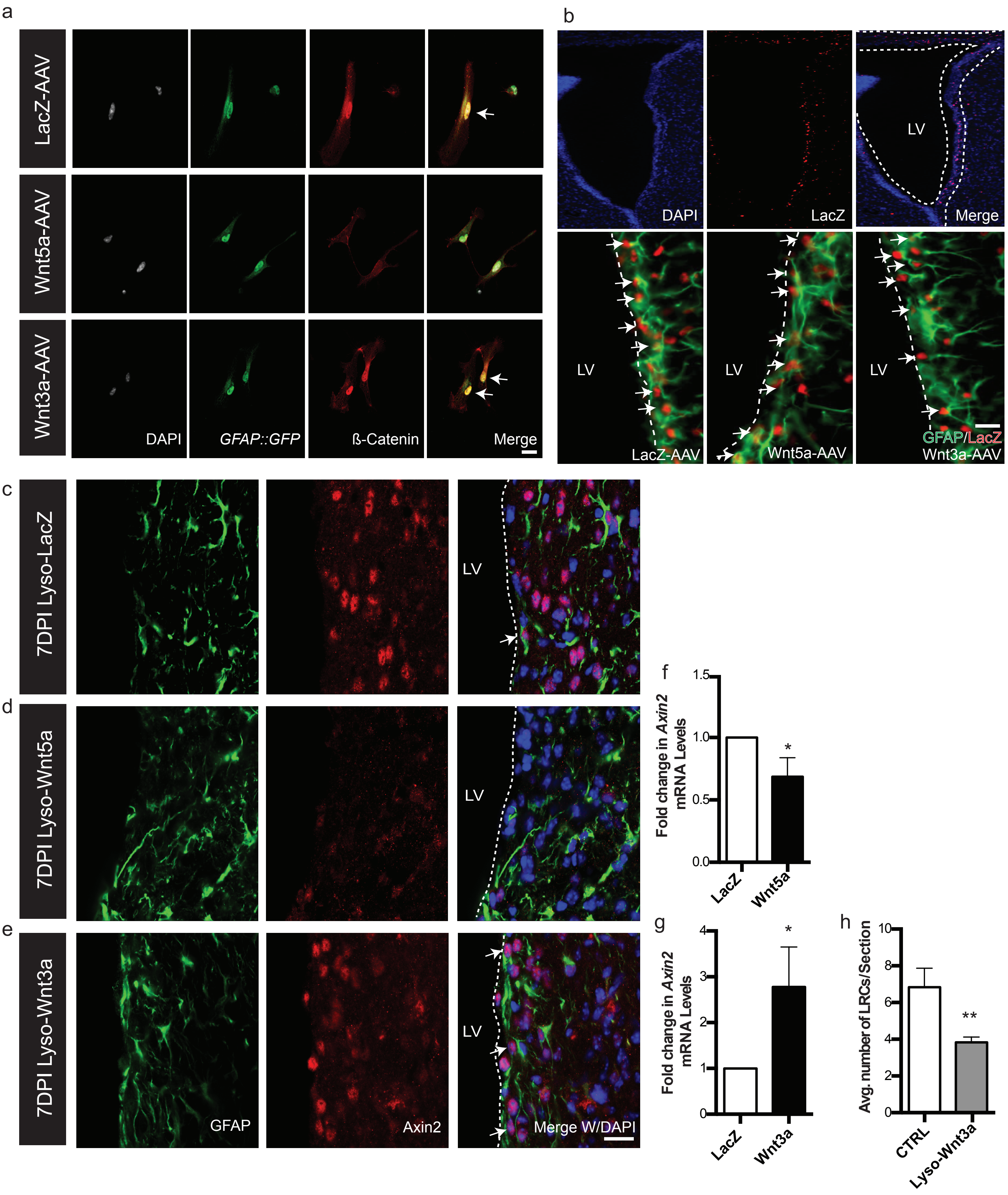

Supplementary Figure 6 Additional analysis of LacZ, Wnt3a, and Wnt5a-AAV Mediated Overexpression in the SVZ

(a) Non-canonical and Canonical Wnt signaling activation as assessed by nuclear ß-catenin translocation in *GFAP::GFP*$^+$ NSCs infected with LacZ, Wnt5a, or Wnt3a-AAVs. Scale bar represents 20μm.

(b) Representative confocal images of SVZ at 7DPI after AAV infection, labeled with anti-LacZ antibodies shows infection of the SVZ. Bottom panels show co-localization of GFAP and LacZ, indicative of AAV infection. Scale bar represents 20μm.

(c-e) Representative images of the SVZ labeled with Anti-Axin2 and GFAP antibodies after infection with LacZ (c), Wnt5a (d) and Wnt3a-AAV (e). A decrease in GFAP$^+$Axin2$^+$ cells in the Wnt5a-AAV infected SVZ (d) and an increase in the same population in the Wnt3a-AAV infected SVZ (e; indicated by arrows) can be observed. Scale bar represents 20μm.

(f) *Axin2* mRNA expression in the SVZ at 7DPI after Wnt5a-AAV infection.

(g) *Axin2* mRNA expression in the SVZ at 7DPI after Wnt3a-AAV infection.

(h) Quantification of BrdU label retaining cells at 7DPI after Wnt3a-AAV infection of the SVZ. (Total number of cells counted CTRL:126; Lyso-Wnt3a:69).

*$p<0.05$, **$p<0.01$; error bars represent mean±s.e.m. n=3 independent experiments for (f-h).

Supplementary Figure 7

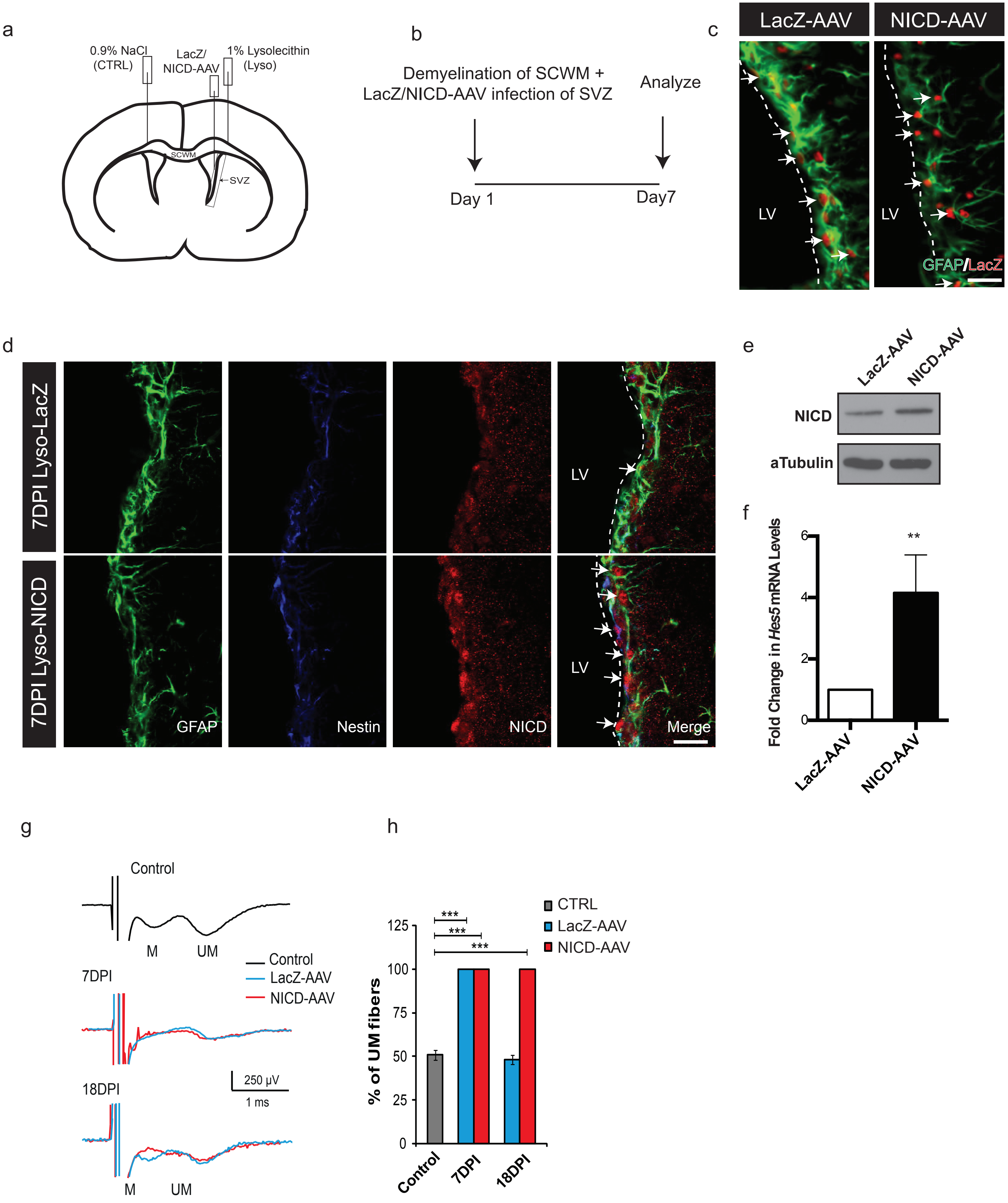

Supplementary Figure 7 Forced Induction of Notch Signaling in the SVZ During Demyelination

(a-b) Experimental paradigm and analysis timeline for forced induction of Notch signaling in the SVZ during demyelination. LacZ (control) or NICD-AAV (activated Notch) were injected into the ventricle, and lysolecithin was injected into corpus collosum to induce demyelination.

(c) Representative confocal images of the SVZ at 7DPI after AAV infection labeled with anti-LacZ and Anti-GFAP antibodies show co-localization of GFAP and LacZ, indicative of efficient AAV infection (arrows). Scale bar represents 20μm.

(d) Representative confocal images of coronal brain sections at 7DPI after AAV infection labeled with anti-GFAP, Nestin, and NICD antibodies show an increase in $GFAP^+Nestin^+NICD^+$ cells after NICD-AAV infection (indicated by arrows). Scale bar represents 20μm.

(e) Immunoblot showing an increase in NICD levels in SVZ protein lysates after infection with NICD-AAV, as compared to LacZ-AAV.

(f) *Hes5* mRNA levels are increased in the SVZ after NICD-AAV infection, represented as fold change over the *Hes5* mRNA levels in the LacZ-AAV infected SVZ.

(g-h) CAP analysis at 7DPI and 18DPI; blue traces show the CAP recordings from the LacZ infected animals and red traces show the recordings from the NICD infected animals. M = myelinated fibers, UM = unmyelinated fibers. At 7DPI both the animals do not show the peak corresponding to myelinated fibers. At 18 DPI, a CAP peak was observed pertaining to remyelinated fibers from LacZ animals, but this was not observed in NICD animals. **$p<0.01$, ***$p<0.001$; error bars represent mean±s.e.m. n=3 for (f) and n=16-19 for (g-h); n=independent experiments.

Supplementary Figure 8

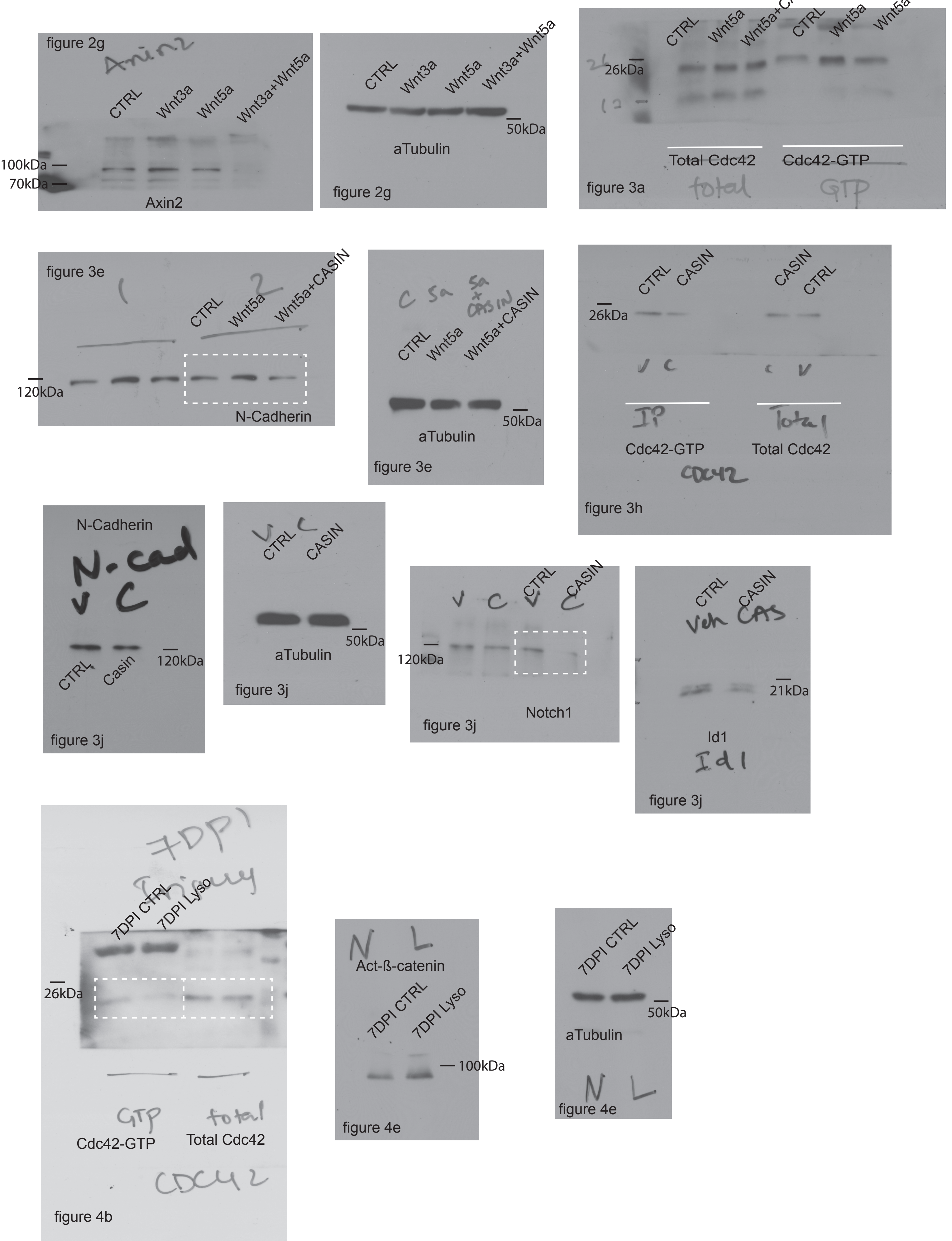

Supplementary Figure 8 Uncropped western blot images from figures 2, 3, and 4.

Supplementary Figure 9

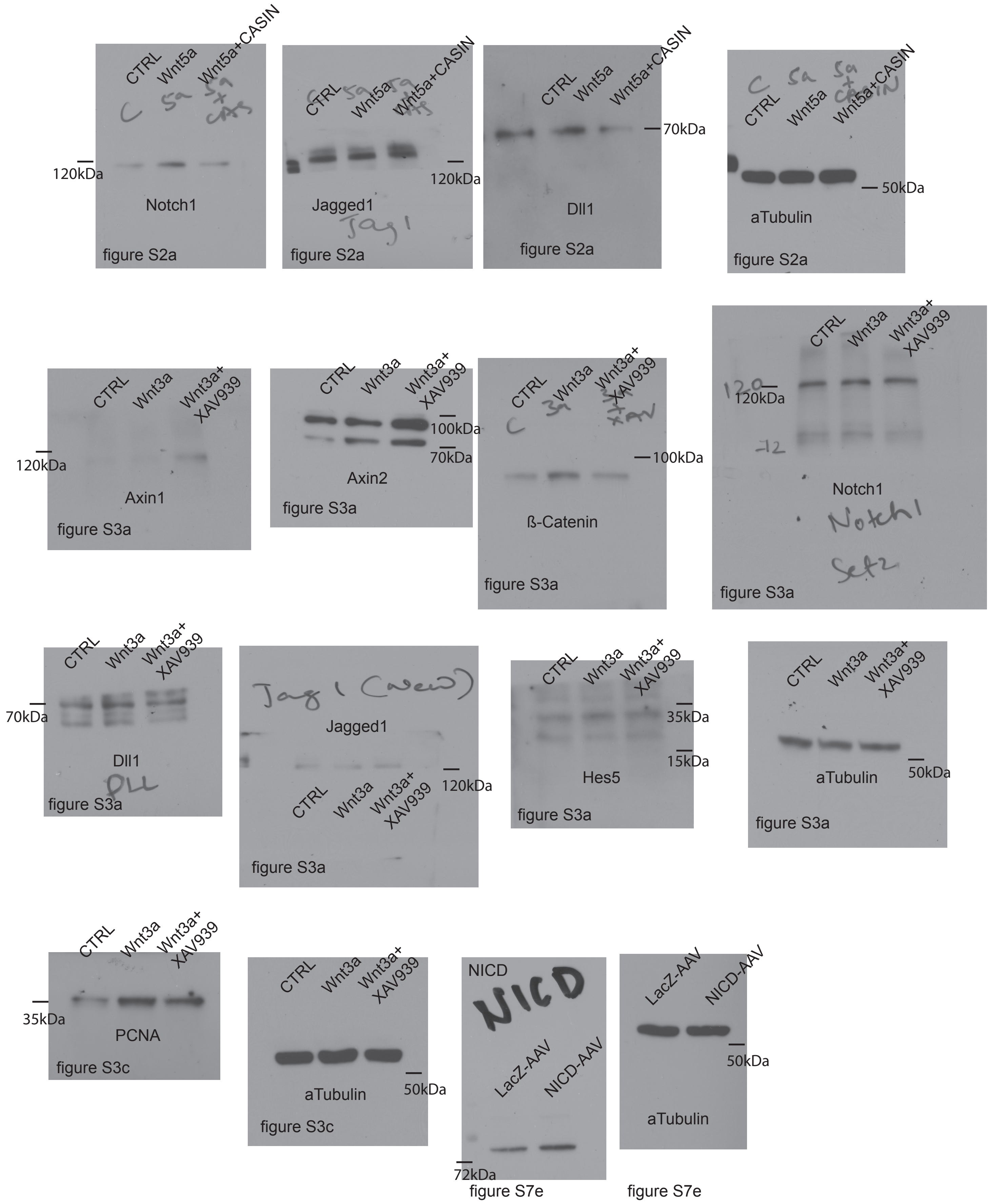

Supplementary Figure 9 Uncropped western blot images from supplementary figures 2, 3, and 7.
