# Supplementary material for: Non-canonical Wnt signaling regulates neural stem cell quiescence during homeostasis and after demyelination
Source: Nat Commun. 2018 Jan 2;9:36. doi: 10.1038/s41467-017-02440-0 (PMC5750230; doi:10.1038/s41467-017-02440-0)
Supplement: Supplementary file 3 — Description of Additional Supplementary Files [file 41467_2017_2440_MOESM3_ESM.pdf]

Description of Supplementary Files

File Name: Supplementary Data 1
Description: Complete list of all protein hits from the proteomics screen and the reactome pathway analysis data.

File Name: Supplementary Data 2
Description: Ct values for the qPCR data presented in Figure 2.

File Name: Supplementary Data 3
Description: List of all the primary antibodies and primer sequence information.
